# Clinicopathological correlations of endometrioid and clear cell carcinomas in the uterus and ovary

**DOI:** 10.1097/MD.0000000000035301

**Published:** 2023-09-15

**Authors:** Hidemi Mori, Haruto Nishida, Takahiro Kusaba, Kazuhiro Kawamura, Yuzo Oyama, Tsutomu Daa

**Affiliations:** a Department of Diagnostic Pathology, Faculty of Medicine, Oita University, Yufu, Japan.

**Keywords:** clinicopathological correlation, differential expression, endometriosis, hypoxia, immunostaining

## Abstract

Endometrioid carcinoma (EC) and clear cell carcinoma (CC) are associated with endometrial tissue hyperplasia and endometriosis, and they occur in the endometrium and ovaries. However, detailed differences between these tumors based on immunostaining are unclear; therefore, in this study, we aimed to analyze the clinicopathological correlations between these tumors using immunostaining and to develop new treatments based on histological subtypes. Immunohistochemistry was used to investigate differentially expressed hypoxia-associated molecules (hypoxia-inducible factor-1 subunit alpha [HIF-1α], forkhead box O1, prostate-specific membrane antigen, signal transducer and activator of transcription 3 [STAT3], hepatocyte nuclear factor 1β [HNF-1β], aquaporin-3, and vimentin [VIM]) between these carcinomas because of the reported association between CC and ischemia. Immunostaining and clinicopathological data from 70 patients (21 uterine endometrioid carcinomas [UECs], 9 uterine cell carcinomas, 20 ovarian endometrioid carcinomas [OECs], and 20 ovarian cell carcinomas [OCCs]) were compared. HIF-1α and prostate-specific membrane antigen expression levels were higher in UEC and OCC than in uterine cell carcinomas and OEC. STAT3 was slightly overexpressed in UEC. Additionally, forkhead box O1 expression was either absent or significantly attenuated in all ECs. VIM and AQ3 were highly expressed in UEC, whereas HNF-1β expression was higher in OCC. UEC, OEC, and OCC were more common in the uterine fundus, left ovary, and right ovary, respectively. Ovarian endometriosis was strongly associated with EC. Our findings suggest that UEC and OCC share a carcinogenic pathway that involves HIF-1α induction under hypoxic conditions via STAT3 expression, resulting in angiogenesis. Furthermore, the anatomical position of carcinomas may contribute to their carcinogenesis. Finally, aquaporin-3 and VIM demonstrate strong potential as biomarkers for UEC, whereas HNF-1β expression is a crucial factor in CC development. These differences in tumor site and histological subtypes shown in this study will lead to the establishment of treatment based on histological and immunohistological classification.

## 1. Introduction

Endometrial and ovarian tumors can appear with various histologies, such as endometrioid carcinoma (EC) and clear cell carcinoma (CC), which are generally associated with endometriosis.^[1]^ The prevalence of ovarian endometriosis is approximately 17–65%, while that of endometriosis-associated neoplasm is approximately 1%.^[1]^ The most common neoplasms are EC and CC; these tumors account for 10% of the ovarian carcinomas.^[2]^ EC and CC do not differ in morphology regardless of their occurrence in the endometrium or ovaries.^[3–6]^ Recently, these genetic alterations are becoming apparent, such as *POLE* mutation, *MSH6, PMS2*, and *p53* in uterine endometrioid carcinoma (UEC).^[3]^ Furthermore, in ovarian EC (OEC) and CC (OCC), these tumors have similar molecular alterations, such as *PIK3CA, ARID1a*, and microsatellite instability/mismatch repair protein.^[2]^ UEC has the same genetical alterations as atypical endometrial hyperplasia, and atypical endometrial hyperplasia is considered a precancerous lesion.^[3,7]^ However, no detailed immunostaining-based observations for tumorigenesis have been made to examine the differences between ovarian and endometrial tumors. No difference exists in therapeutic agents based on tumor site or histologic subtype.^[3–10]^ We can contribute to developing new drugs based on histological subtypes if these tumors’ tumorigenesis and tumor-specific proteins can be clarified. Thus, in this study, we aimed to perform immunostaining to characterize the expression profiles of hypoxia-related molecules (i.e., hypoxia-inducible factor-1 subunit alpha [HIF-1α] and prostate-specific membrane antigen [PSMA]) and molecules specific to endometrial or ovarian cancer (i.e., vimentin [VIM] and aquaporin-3 [AQP3]) in these cancers because OCC is related to hypoxia.^[4]^ Moreover, the relationship between anatomical tumor site and endometriosis was analyzed to investigate the tumor microenvironment and its relationship with tumorigenesis. The findings of this study may serve as a reference for future biomarker identification to aid differential diagnoses of ovarian and endometrial tumors if the site of tumor origin and histological type reveals differences.

## 2. Materials and methods

### 2.1. Patients and clinicopathological data

In this study, we analyzed 70 cases of endometrial and ovarian tumors resected at Oita University Hospital (Yufu, Japan) between January 2003 and April 2021. These included 21 UECs, 9 uterine clear cell carcinomas (UCCs), 20 OECs, and 20 OCCs. Twenty cases were collected from each of the new cases; however, 9 cases were collected in UCCs because there were not many UCC cases. Data, including patient age, tumor location and size, and endometriosis status, were obtained from clinical and/or pathological records. The tumor location was determined macroscopically. This study was performed according to the principles of the Declaration of Helsinki (2013). The institutional ethics committee and review board of Oita University approved this study (approval number 2121). Owing to the retrospective nature of this study, the informed consent requirement was waived; patients could choose to decline the use of their information in any research project at admission.

### 2.2. Histology and immunohistochemistry (IHC)

Formalin-fixed paraffin-embedded tissue blocks were sliced to 4 μm thickness for hematoxylin and eosin and IHC staining. We confirmed the histological diagnoses according to World Health Organization classifications.^[3–6]^ Sections were deparaffinized in xylene and rehydrated in graded alcohol before IHC staining. Endogenous peroxidase activity was blocked via incubation in 3% hydrogen peroxidase for 20 minutes at room temperature (25 ℃). Antigens were retrieved via autoclaving (in citrate buffer; pH 6.0 or 9.0). Subsequently, slides were incubated with primary antibodies (Table 1).

**Table 1:** Primary antibodies used in immunohistochemical staining.

Antibody	Citrate buffer	Dilution	Incubation temperature and time	Additional conditions	Source
HIF-1α	pH 9	1:100	25°C, 30 min	No	Novus
FOXO1	pH 6	1:50	4°C, overnight	Simple stain	Cell signaling
PSMA	pH 9	-	4°C, overnight	No	Dako
STAT3	pH 9	1:500	4°C, overnight	Simple stain	Abcam
HNF-1β	pH 6	1:500	25°C, 30 min	No	ProteinTech
Vimentin	–	–	4°C, overnight	No	Nichirei
Aquaporin-3	pH 9	1:200	4°C, overnight	Simple stain	Abcam

FOXO1 = forkhead box O1; HIF-1α = hypoxia-inducible factor-1 subunit alpha; HNF-1β = hepatocyte nuclear factor-1 beta; PSMA = prostate-specific membrane antigen; STAT3 = signal transducer and activator of transcription 3.

Immunoreactivity was visualized using a streptavidin-labeled biotin-peroxidase complex system (Nichirei, Tokyo, Japan). Two independent observers (HM and HN) evaluated each IHC sample to identify positive reactions. However, these immunohistological markers were not usually clearly defined; therefore, we defined positivity as more than 10% of tumor cells expressing estrogen receptor and human epidermal growth factor receptor 2.^[11,12]^ Additionally, samples with more positive tumor cells were categorized into 2 groups based on 50% or more positivity. Reactivity was scored based on colorimetric intensity and the positive cell population. Staining was considered positive if immunoreactivity was observed in ≥50% (nuclear: signal transducer and activator of transcription 3 [STAT3] and hepatocyte nuclear factor [HNF]-1β; cytoplasmic: VIM, and PSMA) or ≥10% (cell membrane: AQP3 and nuclear: HIF-1α) of the tumor. Forkhead box O1 (FOXO1) staining was considered positive if nuclear immunoreactivity was either absent or reduced.

### 2.3. Statistical analyses

Statistical analyses were performed using Student *t*-tests to examine the associations for each biomarker. In all cases, statistical significance was set at *P* < .05.

## 3. Results

**Figure 1.**
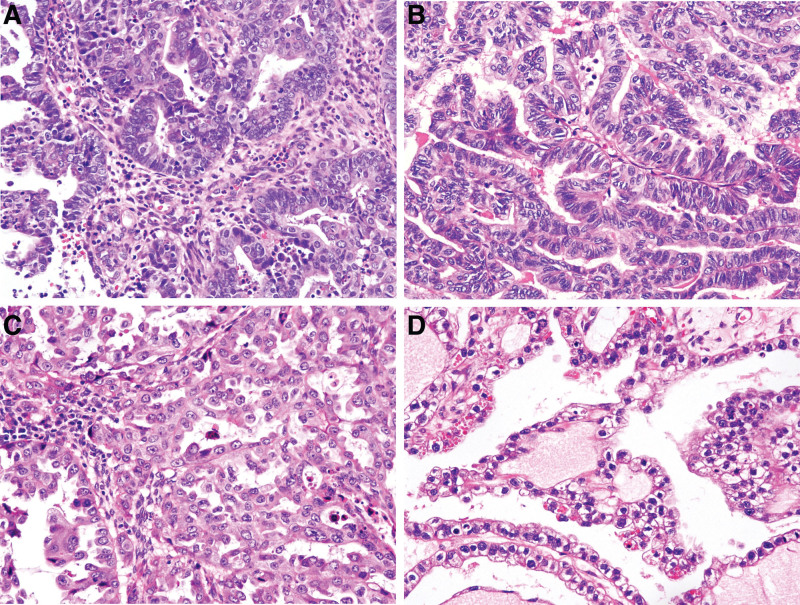
Endometrioid carcinoma of the uterus (A) and ovary (B) and clear cell carcinoma of the uterus (C) and ovary (D) share similar morphological features.

**Figure 2.**
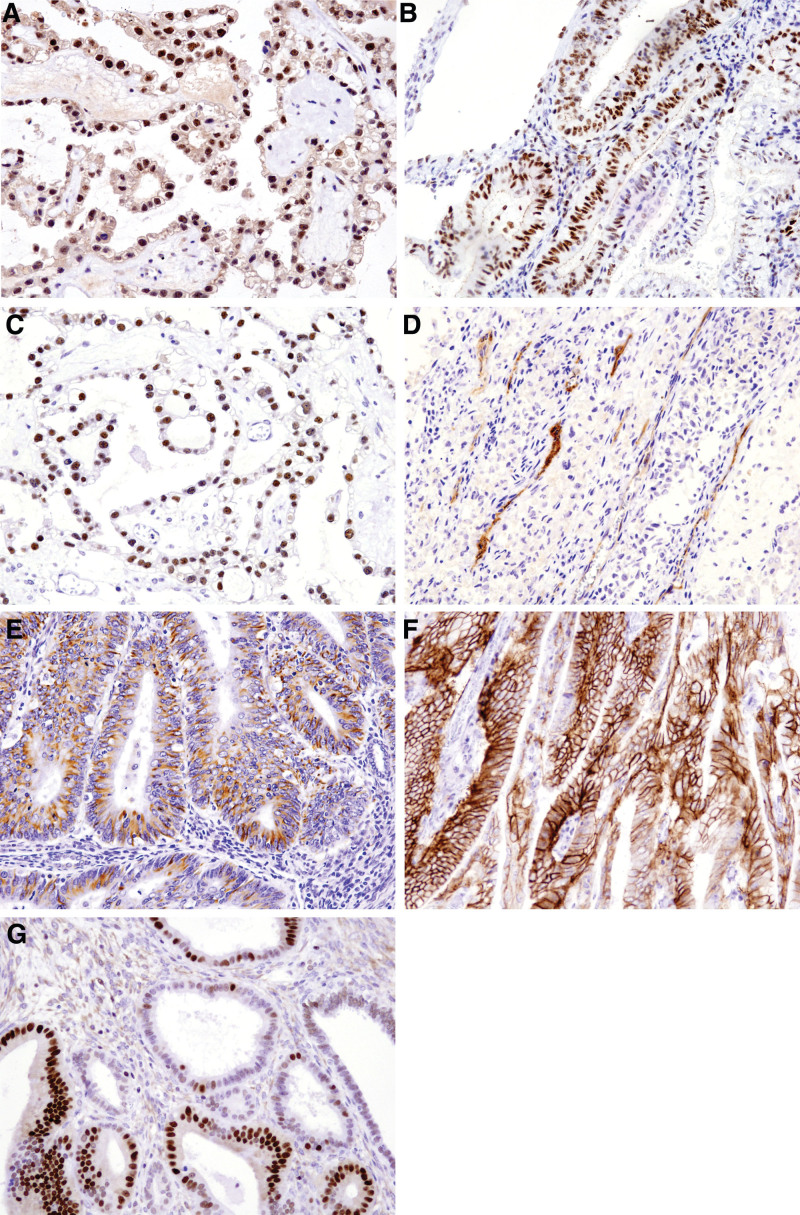
HIF-1α (×40) (A), STAT3 (×40) (B), and HNF-1β (×40) (C) expression in tumor nuclei. The neovascular cytoplasm of the tumor appears positive for PSMA (D) (×40). The tumor cytoplasm also appears positive for VIM (E) and AQP3 (F). FOXO1 (G) (×40) expression appears either absent or reduced in all cancers. AQP3 = aquaporin-3; FOXO1 = forkhead box O1; HIF-1α = hypoxia-inducible factor-1 subunit alpha; HNF-1β = hepatocyte nuclear factor-1 beta; PSMA = prostate-specific membrane antigen; STAT3 = signal transducer and activator of transcription 3; VIM = vimentin.

In this study, uterine and ovarian EC and CC had similar morphological features; however, cystic and large glandular structures were more prominent in ovarian tumors (Fig. 1A–D). IHC staining showed that HIF-1α and PSMA expression was higher in UEC and OCC than in UCC (Fig. 2A–G; Tables 2 and 3) and tended to be higher than that in OEC. STAT3 was slightly overexpressed in UEC compared with that in other tumors (91%). FOXO1 expression was either absent or significantly reduced in EC (*P* < .05) (Table 2). VIM and AQP3 expression was significantly higher in UEC, whereas hepatocyte nuclear factor 1β [HNF-1β] expression was significantly higher in CC (especially OCC) than in EC (Table 3). Additionally, patients diagnosed with OCC were younger than those diagnosed with other carcinomas.

**Table 2:** Immunohistochemical results of STAT3, HIF-1α, PSMA, and FOXO1.

Antibody	UEC	OEC	UCC	OCC
STAT3	90% (19/21)	50% (10/20)	67% (6/9)	75% (15/20)
HIF-1α	90% (19/21)	20% (4/20)	33% (3/9)	80% (16/20)
PSMA	86% (18/21)	70% (14/20)	22% (2/9)	90% (18/20)
FOXO1*	**100% (21/21**)	**100% (20/20**)	78% (7/9)	15% (3/20)

The BOLD number was significant (*P* < .05).

FOXO1 = forkhead box O1; HIF-1α = hypoxia-inducible factor-1 subunit alpha; OCC = ovarian clear cell carcinoma; OEC = ovarian endometrioid carcinoma, PSMA = prostate-specific membrane antigen; STAT3 = signal transducer and activator of transcription 3, UCC = uterine clear cell carcinoma, UEC = uterine endometrioid carcinoma.

* FOXO1 expression was considered positive if immunoreactivity was lost or reduced.

**Table 3:** Immunohistochemical results of vimentin, aquaporin-3, and HNF-1β.

	UEC	OEC	UCC	OCC
Vimentin	**90% (19/21**)	25% (5/20)	33% (3/9)	0% (0/20)
Aquaporin-3	**62% (13/21**)	15% (3/20)	0% (0/9)	0% (0/20)
HNF-1β	19% (4/21)	5% (1/20)	**78% (7/9**)	**100% (20/20**)

The BOLD number was significant (*P* < .05).

HNF-1β, hepatocyte nuclear factor-1 beta; OCC = ovarian clear cell carcinoma; OEC = ovarian endometrioid carcinoma, UCC = uterine clear cell carcinoma, UEC = uterine endometrioid carcinoma.

UECs are commonly found in the fundus or corpus or diffusely distributed in the endometrium (Table 4). In this study, UECs were found diffusely or in the fundus. All ECs in the fundus were relatively small (<5 cm). Regarding ovarian tumors, OECs and OCCs were predominantly located in the left and right ovaries, respectively. All OCCs were larger than OECs, except for one (>6 cm). The frequency of endometriosis-associated complications was slightly higher in the OEC group than in the OCC group (Table 4).

**Table 4:** Tumor clinical data.

	UEC	OEC	UCC	OCC
Age (average, in years)	62.9	58.5	66.9	54.0
Tumor location	Fundus: 10 Corpus: 1 Diffuse: 10	Left: 14 Right: 6	Fundus: 4 Corpus: 2 Diffuse: 3	Left: 7 Right: 13
Endometriosis	–	Left: 8 Right: 2	–	Left: 4 Right: 4
Tumor size (cm)	4.2 (0.7–10.5)	7.4 (1.0–20)	4.4 (2.5–5.5)	11.9 (2–27)

OCC = ovarian clear cell carcinoma, OEC = ovarian endometrioid carcinoma, UCC = uterine clear cell carcinoma, UEC = uterine endometrioid carcinoma.

## 4. Discussion

HIF-1α binds HIF-1β to form HIF-1, an intranuclear transcription factor that induces angiogenesis under hypoxic conditions.^[13]^ HIF-1α is also a co-activator of estrogen-induced vascular endothelial growth factor in both normal endometrial tissues and EC.^[14]^ Recently, neovascular PSMA expression has been associated with tumors and hypoxia severity.^[15]^ In this study, HIF-1α and PSMA were overexpressed in UEC and OCC, suggesting that both tumor types are hypoxic, regardless of their primary site or morphological features. Previous research has revealed that HIF-1α is expressed in OCC via the interleukin 6-(IL-6)– STAT3–HIF pathway.^[16]^ Similarly, we found significant STAT3 and HIF-1α expression in OCC. PSMA, STAT3, and HIF-1α were overexpressed in UEC, suggesting that angiogenesis is induced via the IL-6–STAT3–HIF-1 pathway, comparable to that in OCC. These data indicate that under hypoxic conditions, HIF-1α overexpression is related to STAT3-mediated angiogenesis promotion, which mediates UEC and OCC carcinogenesis and progression.

FOXO1 is a transcription factor expressed in various organs, such as the liver, pancreas, and hypothalamus.^[17]^ It regulates various cellular functions, including cell differentiation and proliferation.^[17]^ FOXO1 mRNA is degraded in UEC, downregulating intranuclear FOXO1 expression.^[18]^ FOXO1 downregulation also decreases the expression of progesterone receptors and is linked to ovarian cancer.^[19]^ HIF-1α-mediated upregulation of vascular endothelial growth factor expression in response to progesterone withdrawal and hypoxia may underlie this phenomenon.^[20]^ In this study, FOXO1 expression was either absent or reduced in all UECs and OECs. The small number of UCC cases limited our observations; however, UECs (particularly early lesions) were found more frequently in the uterine fundus than UCCs, which is likely because the fundus is more hypoxic than other parts of the uterus. In combination with the present data on HIF-1α overexpression, our FOXO1 data support a relationship between UEC and hypoxia.

**Figure 3.**
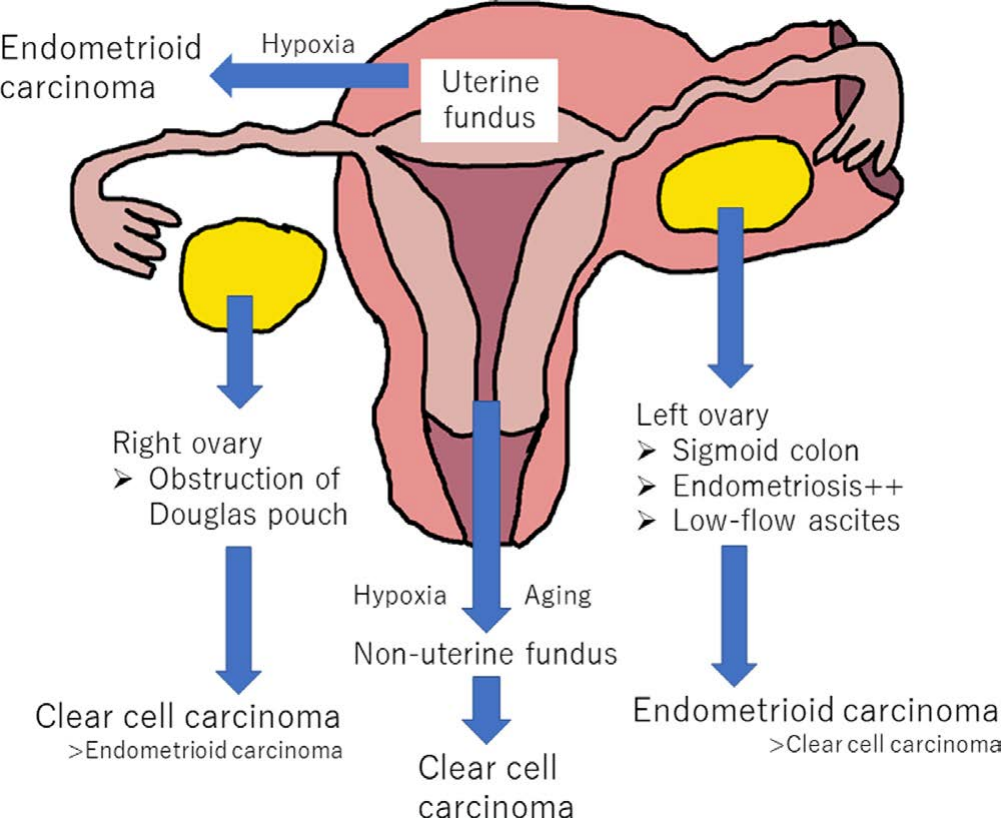
Schema of the histological tumor differences and tumor locations. The prevalence of endometriosis in the left ovary is explained by the presence of the sigmoid colon, which delays the spread of retrograde menstrual blood, resulting in weak and slowed fluid flow in the left hemipelvis. Endometriosis of the right ovary is more severe than that of the left ovary, and obliteration of the pouch of Douglas occurs more frequently in the right ovary. This may increase the severity of hypoxia, leading to higher ovarian clear cell carcinoma occurrence in the right ovary. Uterine endometrioid carcinomas (particularly early lesions) were found more frequently in the uterine fundus than uterine clear cell carcinomas, which is likely because the fundus is more hypoxic than other parts of the uterus.

OEC and OCC occurred more frequently in the left and right ovaries, respectively. Endometriosis was also more common in the left ovary, which is consistent with previous studies.^[21]^ The prevalence of endometriosis in the left ovary is explained by the presence of the sigmoid colon, which delays the spread of retrograde menstrual blood, resulting in weak and slowed fluid flow in the left hemipelvis.^[21]^ Accordingly, OECs originate from the epithelium of endometriotic tissue and are associated with FOXO1 expression loss, similar to UECs. Endometriosis of the right ovary is more severe than that of the left ovary, and obliteration of the pouch of Douglas occurs more frequently in the right ovary.^[22]^ This may increase the severity of hypoxia, leading to higher OCC occurrence in the right ovary. Based on these hypotheses, tumorigenesis and histological subtypes are shown in Figure 3. In UEC, some biochemical and IHC biomarkers, such as TRMP7, may also be used to predict cancer progression in endometrial hyperplasia or are related to cancer progression.^[7]^

Given its specific expression in OCC, HNF-1β is currently used in differential diagnosis as an OCC biomarker.^[23]^ This study confirmed that HNF-1β is overexpressed in OCC and UCC compared with the expression in OEC and UEC. HNF-1β is a transcription factor that protects cells from oxidative stress by inducing antioxidant protein expression.^[24]^ Furthermore, HNF-1β upregulation activates STAT3 and nuclear factor kappa B signaling to promote IL-6 and IL-8 production.^[25]^ Therefore, HNF-1β upregulation may activate IL-6–STAT3–HIF signaling, particularly in OCC. HNF-1β was also overexpressed in CCs, suggesting that HNF-1β is a crucial factor in CC development, with severe hypoxia triggering HNF-1β methylation. OCC staining was highly positive for HIF-1α. In this study, UCC occurred in older patients; HNF-1β overexpression is associated with aging-related or long-term decreases in DNA methylation that are more severe than EC-related decreases.^[26,27]^

VIM is specifically expressed in UEC and is a biomarker for the differential diagnosis of ECs. Our results confirmed that VIM and AQP3 are overexpressed in UEC. Recent research has reported AQP3 expression in UEC; however, no report has compared its expression across histological subtypes of other carcinomas, such as ovarian cancer.^[28]^ Some studies have also described a relationship between VIM and AQP3 expression and hypoxia.^[29,30]^ We found that AQP3 overexpression is a potential biomarker for the differential diagnosis of ECs. Furthermore, because of its specificity to UEC, AQP3 expression may indicate primary UEC if the tumor is histologically diagnosed as EC.

The limitations of this study include it being a single institute study with a small number of cases. However, the new insight into uterine and ovarian EC and CC could serve as a reference for future research and treatment.

In conclusion, UEC and OCC showed high HIF-1α, STAT3, and PSMA expression, indicating that both cancer types share developmental and progression pathways. Specifically, hypoxia induces HIF-1α expression via the STAT3 pathway, resulting in angiogenesis. FOXO1 expression was either absent or significantly reduced in all UECs and OECs, suggesting that it is a crucial factor in EC development. In addition, specific anatomical tumor sites were linked to specific carcinomas. OECs occurred in the left ovary, OCCs in the right ovary, and UECs in the uterine fundus. These locational features correspond with the associated pathological or anatomical characteristics. For instance, OECs are oriented toward the left ovaries because they are associated with endometriosis, which is more common in the left ovaries. Therefore, future studies should evaluate whether these anatomical observations are clinically relevant—for instance, whether they can be used to diagnose these cancers. Finally, based on our findings, AQP3 and VIM can be used as biomarkers for the differential diagnosis of UEC, whereas HNF-1β expression is crucial in CC development. The differences in tumorigenesis between tumor locations and histological subtypes may be valuable in tumor treatment and drug selection.

## Author contributions

**Conceptualization:** Hidemi Mori, Haruto Nishida.

**Data curation:** Hidemi Mori, Haruto Nishida, Takahiro Kusaba, Kazuhiro Kawamura, Yuzo Oyama, Tsutomu Daa.

**Formal analysis:** Hidemi Mori, Haruto Nishida.

**Investigation:** Hidemi Mori, Haruto Nishida.

**Methodology:** Hidemi Mori, Haruto Nishida.

**Project administration:** Haruto Nishida, Tsutomu Daa.

**Resources:** Haruto Nishida.

**Supervision:** Haruto Nishida, Tsutomu Daa.

**Validation:** Haruto Nishida.

**Visualization:** Haruto Nishida.

**Writing – original draft:** Hidemi Mori, Haruto Nishida.

**Writing – review & editing:** Haruto Nishida, Takahiro Kusaba, Kazuhiro Kawamura, Yuzo Oyama, Tsutomu Daa.
